# Why Scientists Must Engage: Environmental Health in Times of Armed Conflict

**DOI:** 10.3389/ijph.2025.1609353

**Published:** 2025-12-17

**Authors:** Manolis Kogevinas, Marianthi-Anna Kioumourtzoglou, Amira Aker, Anass Houdou, Stephanie Grady, Aziza Menouni, Youssef Oulhote, Beate Ritz, Ellen M. Wells, Wael K. Al-Delaimy

**Affiliations:** 1 Instituto Salud Global (ISGlobal), Barcelona, Spain; 2 CIBER Epidemiologia y Salud Pública (CIBERESP), Madrid, Spain; 3 Department of Epidemiology, School of Public Health, Brown University, Providence, RI, United States; 4 Institute at Brown University for Environment and Society, Brown University, Providence, RI, United States; 5 Department of Epidemiology, School of Public Health, Boston University, Boston, MA, United States; 6 Herbert Wertheim School of Public Health and Human Longevity Science, University of California San Diego, La Jolla, CA, United States; 7 Department of Environmental Health, School of Public Health, Boston University, Boston, MA, United States; 8 Environment & Health Unit, Department of Public Health & Primacy Care, Katholieke Universiteit Leuven, Leuven, Belgium; 9 Research Department, Thriving Lab, Casablanca, Morocco; 10 Departments of Environmental Medicine and Global Health and Health Systems Design, Icahn School of Medicine, Mt Sinai, New York, NY, United States; 11 Departments of Environmental Health Sciences and Epidemiology, UCLA Fielding School of Public Health, Los Angeles, CA, United States; 12 School of Health Sciences and Department of Public Health, Purdue University, West Lafayette, IN, United States

**Keywords:** environmental health, armed conflicts, accountability and justice, displacement and health, humanitarian health

## Introduction

Armed conflict is a profound determinant of health. Beyond direct violence, wars devastate the environmental foundations on which public health depends, including clean air, safe water, fertile soil, functioning infrastructure, and loss of qualified health professionals [1]. Armed conflict consequences persist long after hostilities end, silently shaping and expanding disease, displacement, and inequity for generations. Yet, systematic scientific engagement with conflict-related environmental health dimensions has been limited.

This commentary is part of a broader series in the *International Journal of Public Health* addressing the health challenges of displaced and conflict-affected populations. In his editorial, Saracci highlights how forced displacement, driven largely by armed conflict, has become a defining demographic and public health challenge of our era, calling for stronger epidemiological engagement with the structural and environmental determinants of displacement [2]. His framing underscores the urgency of examining not only the social but also the environmental health consequences of contemporary conflicts, which this commentary takes up.

There is a growing recognition among ISEE members that armed conflict destroys essential environmental and public health infrastructure, creating cascading risks that can endure for years. This awareness was triggered by the environmental devastation following the Russian invasion of Ukraine and has intensified over the past 2 years with the unprecedented scale of destruction in Gaza resulting from the bombing and Israeli military campaign. In response, the newly formed ISEE Armed Conflict and Environmental Health Special Interest Group (SIG) aims to mobilize researchers, strengthen collaborations, and promote accountability for the environmental health consequences of armed conflict.

## Expanding the Definition of Conflict

Traditional conflict metrics, e.g., those used in political databases, focus on battle-related deaths and injuries or state-based confrontations. While useful for quantifying violence, they obscure the broader and longer-term consequences for ecosystems and civilian health during and after conflict, including the lasting mental and physical harms experienced by soldiers and the subsequent impacts on civil society, as seen historically among veterans exposed to substances such as Agent Orange [3].

The ISEE SIG defines conflict, for the purposes of its work, as any sustained situation of organized violence, occupation, or systemic insecurity that disrupts environmental systems and exposes populations to environmental health risks. This framing includes the collapse of water and sanitation systems, contamination of soil and groundwater, release of toxic and hazardous waste, unsafe and traumatic exposures to noise and vibration, and the environmental pressures linked to displacement and resource scarcity. It extends across the full conflict continuum, from acute fighting to post-conflict recovery, during which cumulative exposures continue to affect human and ecological wellbeing for generations. It also acknowledges transboundary environmental health consequences that can affect neighbouring areas, ecosystem integrity, and regional wellbeing.

This broader lens recognizes that environmental degradation is both a driver and a consequence of conflict, and that safeguarding environmental health is essential to humanitarian protection, peacebuilding, and reconstruction. Furthermore, preexisting environmental or health-related conditions may exacerbate some environmental health consequences of conflict.

## Environmental Health as a Humanitarian Imperative

In most modern conflicts, civilian populations face both violence and environmental collapse. Environmental health protection, therefore, is not a peripheral concern but a core component of humanitarian action. This aligns with WHO guidance on environmental health in emergencies, which emphasizes the centrality of environmental services to public health protection [4]. Yet, structured monitoring of environmental exposures in conflict zones remains scarce. Data collection is fragmented, often driven by humanitarian agencies or investigative journalists rather than sustained scientific networks. As a result, populations at risk remain largely invisible in the global environmental health agenda.

Scientific societies such as ISEE can help change this. By pooling expertise in exposure science, epidemiology, toxicology, and risk assessment, they can bring rigor, transparency, and comparability to documenting environmental health impacts of conflict and inform mitigation and recovery interventions.

## The ISEE Initiative: Science, Ethics, and Action

The newly established ISEE *SIG* seeks to serve both the scientific community and affected populations through four main lines of work.

### Capacity Building and Partnerships

The group aims to strengthen collaborations with local researchers and institutions in conflict-affected areas, promoting training, mentorship, and remote or *in-situ* technical support for environmental and health assessments. Partnerships with NGOs, UN agencies, and local governments will be key to ensuring sustainability and mutual trust.

### Surveillance and Documentation

Documentation of environmental exposures, including air, water, soil, radiation, and waste monitoring, are essential. The group will support efforts to develop standardized methods and indicators of the “environmental health burden of conflict,” and compile case studies across regions. Such data can help reveal common patterns, from infrastructure collapse to environmental contamination, and guide remediation and reconstruction priorities. The destruction of the Kakhovka Dam, for example, produced extensive hydrological and ecological impacts documented in recent scientific assessments [5]. Just like we investigate proximal and distal causes in epidemiology and identify exposure sources, we also need to identify the sources of environmental damage in a conflict and the responsible parties.

### Research and Methodological Development

Conflict conditions demand innovative study designs and ethical protocols while being responsive. In collaboration with existing key conflict-related research groups, the ISEE SIG group will foster methodological guidance on how to conduct environmental health research under insecurity and pressure, including community engagement, remote sensing, and protection of local collaborators. Such guidance will help researchers collect valid data without compromising safety or integrity.

### Ethics, Justice, and Accountability

Conflict invariably amplifies power asymmetries. The SIG commits to scientific objectivity grounded in evidence and ethical integrity, recognizing that impartiality must not become indifference or, worse, complicity [6].

Environmental epidemiologists, toxicologists, and exposure scientists can and should contribute to accountability mechanisms, from documenting damage to supporting claims for reparations or environmental restoration. While the resolution of such issues requires legal and policy action, our scientific expertise is crucial to inform and substantiate these processes by documenting impacts and providing evidence essential for fair accountability.

## Why Scientific Societies Must Act

Public and environmental health are inherently political; using science to highlight suffering from environmental destruction is a scientific and moral responsibility. Scientific associations offer both the collective legitimacy and the protective structure needed for scientists to engage in politically sensitive fields. They can act as boundary institutions, linking research with policy, humanitarian practice, and affected communities.

Moreover, scientific communities can amplify voices from conflict-affected regions, supporting inclusion, safety, and equity in global scientific discourse. For early-career researchers and local scientists, such networks provide visibility and access to otherwise-inaccessible resources.

## From Documentation to Recovery

Post-conflict reconstruction offers opportunities to equitably integrate health and sustainability principles into rebuilding efforts. This includes evaluating and guiding environmental remediation, soil clean-up, waste management, and water and energy system restoration, while ensuring resilience to future climate and conflict stressors.

By linking recovery to environmental health science, the SIG seeks to promote a vision of *“build back healthier,”* where reconstruction addresses, rather than reproduces, pre-existing vulnerabilities.

## A Call to the Public Health Community

The tools of environmental epidemiology, exposure assessment, geospatial analysis, toxicology, and risk modelling are powerful instruments for understanding and mitigating the consequences of armed conflict. We invite public and environmental health scientific communities to:

- Support systematic monitoring and research on environmental destruction leading to exposures in conflict zones.
- Collaborate equitably with local researchers and communities.
- Promote open data and methodological transparency in documenting environmental damage.
- Integrate environmental health perspectives into humanitarian, peacebuilding, and reconstruction policies.

We encourage funding agencies to support such efforts, recognizing their importance for science, policy, and humanitarian response.

### Conclusion

Armed conflicts reshape environments as profoundly as they reshape societies. Their environmental legacies, including poisoned air and water, destroyed ecosystems, contaminated soil, are public health crises that demand evidence, compassion, and accountability.

By engaging with these challenges, scientists reaffirm their ethical and civic responsibility: to bear witness, document harm, and help rebuild healthier futures. The ISEE *Armed Conflict and Environmental Health SIG* represents a step toward institutionalizing that commitment within our discipline.
